# Probiotic Administration Mitigates Bisphenol A Reproductive Toxicity in Zebrafish

**DOI:** 10.3390/ijms22179314

**Published:** 2021-08-27

**Authors:** Christian Giommi, Hamid R. Habibi, Michela Candelma, Oliana Carnevali, Francesca Maradonna

**Affiliations:** 1 Dipartimento Scienze della Vita e dell’Ambiente, Università Politecnica delle Marche, Via Brecce Bianche, 60131 Ancona, Italy; c.giommi@pm.univpm.it (C.G.); m.candelma@staff.univpm.it (M.C.); 2 Department of Biological Sciences, University of Calgary, Calgary, AB T2N 1N4, Canada; habibi@ucalgary.ca; 3 INBB—Consorzio Interuniversitario di Biosistemi e Biostrutture, 00136 Roma, Italy

**Keywords:** *Danio rerio*, fecundity, reproduction, probiotic, endocrine disruptors

## Abstract

Although the use of bisphenol A (BPA) has been banned in a number of countries, its presence in the environment still creates health issues both for humans and wildlife. So far, BPA toxicity has been largely investigated on different biological processes, from reproduction to development, immune system, and metabolism. In zebrafish, *Danio rerio*, previous studies revealed the ability of environmentally relevant concentrations of this contaminant to significantly impair fertility via epigenetic modification. In addition, several studies demonstrated the ability of different probiotic strains to improve organism health. This study provides information on the role of the probiotic mixture SLAb51 to counteract adverse BPA effects on reproduction. A 28-day trial was set up with different experimental groups: BPA, exposed to 10 µg/L BPA; P, receiving a dietary supplementation of SLAb51 at a final concentration of 10^9^ CFU/g; BPA+P exposed to 10 µg/L BPA and receiving SLAb51 at a final concentration of 10^9^ CFU/g and a C group. Since oocyte growth and maturation represent key aspects for fertility in females, studies were performed on isolated class III (vitellogenic) and IV (in maturation) follicles and liver, with emphasis on the modulation of the different vitellogenin isoforms. In males, key signals regulating spermatogenesis were investigated. Results demonstrated that in fish exposed to the combination of BPA and probiotic, most of the transcripts were closer to C or P levels, supporting the hypothesis of SLAb51 to antagonize BPA toxicity. This study represents the first evidence related to the use of SLAb51 to improve reproduction and open new fields of investigation regarding its use to reduce endocrine disrupting compound impacts on health.

## 1. Introduction

The surrounding environment is contaminated by a broad range of organic pollutants with endocrine-disrupting properties able to alter the endocrine system and cause various health problems by interfering with the organism’s physiology [1,2,3]. It is well known that among them, bisphenol A (BPA), abundantly used in plastic food containers, water bottles, and personal care devices, affects reproduction, in part, by impairing gametogenesis in humans and wildlife [4,5,6,7]. In zebrafish, chronic exposure to 5 µg/L BPA blocked ovulation by deregulating epigenetic mechanism [8,9]. Feeding of BPA-contaminated diet in juvenile seabream led to increased feminization process [10], induced hepatotoxicity, altered lipid metabolism [11], and altered fillet macromolecular composition [12]. There is increasing awareness of the need to come up with strategies to minimize the impact of EDCs. At the present time, wastewater treatment plants cannot completely remove EDCs, which are found in wastewater effluents discharged into the aquatic environment. There is increasing effort to improve EDC management strategies and develop technologies to promote sustainable and environmentally responsible wastewater treatment plants [13]. One promising option for the remediation of EDCs is to select more appropriate microbial communities, microalgae, or fungi to enhance wastewater treatment plants [13]. An additional approach would be to improve the organism’s capacity to minimize adverse actions of contaminants by enhancing the host stress tolerance and immune response. Recent studies suggest that probiotics may improve tolerance to EDC toxicity, as demonstrated by a plethora of studies describing the beneficial effects of probiotic strain administration on different physiological processes [14,15,16,17,18,19,20]. In this context, a recent study demonstrated the ability of *Lactiplantibacillus plantarum* strain to lower BPA toxicity [21], in part, by reducing its biosorption and increasing its biodegradation [22]. Evidence suggests that probiotic strains can differently modulate biological processes and display different modes of action in a sex-specific manner [23]. The present study provides novel information on the ability of SLAb51 to counteract the adverse effects of BPA on reproduction in zebrafish.

## 2. Results

### 2.1. Fertility

Fertility is expressed as the mean ± standard deviation (SD) of fertilized eggs per female per day. Treatments administered did not show significant differences among experimental groups (Control -C-; Bisphenol A -BPA-; Bisphenol A+Probiotic -BPA+P; Probiotic -P-), although a decrease was observed in BPA treated groups compared to the control. The number of collected embryos includes BPA 86.08 ± 37.63, BPA+P 76.33 ± 42.89 and P 96.25 ± 13.73 eggs, compared to C fish (107.75 ± 24.97 eggs).

### 2.2. Gonadal Histological Analysis

**Figure 1:**
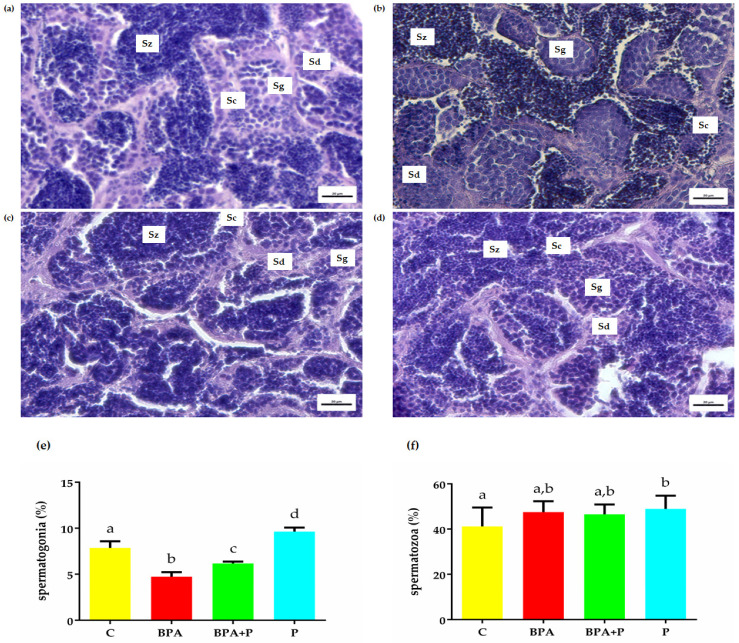
Histological sections of testis: C (**a**), BPA (**b**), BPA+P (**c**), and P (**d**). Eosin-Mayer’s haematoxylin staining. Sg: spermatogonia; Sc: spermatocyte; Sd: spermatid; Sz: spermatozoa. Scale bar: 20 µm. Percentage of zebrafish testicular area occupied by spermatogonia (**e**) and spermatozoa (**f**). Data reported as means ± SEM. Different letters denote statistically significant differences among experimental groups (one-way ANOVA, *p* < 0.05, Dunnett’s multiple comparison test).

The area covered by spermatogonia and spermatozoa was measured in the testis sections obtained from different groups (Figure 1). The spermatogonia number decreased in groups exposed to BPA and increased in testis from P fish. The results demonstrate that P mitigates BPA toxicity since, in the BPA+P group, a significant increase in spermatogonia number was observed compared to BPA. Moreover, P treatment significantly increased the number of spermatozoa compared to C (Figure 1e,f).

**Figure 2:**
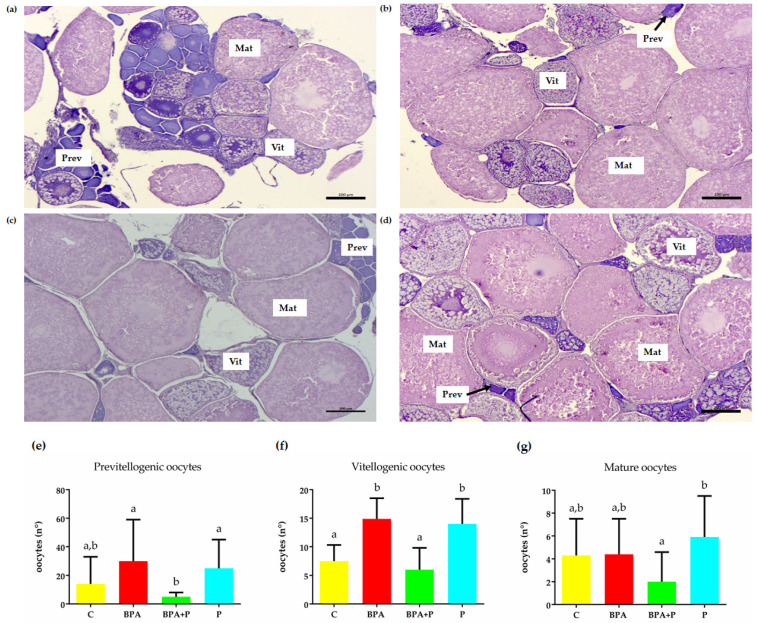
Histological analysis of ovaries from C (**a**), BPA (**b**), BPA+P (**c**) and P (**d**). Ovarian sections show different follicular stages. Eosin and Mayer’s haematoxylin staining. Prev: previtellogenic oocytes; Vit: Vitellogenic oocytes; Mat: mature oocytes. Scale bar: 200 µm. Percentage of different follicle classes (**e**–**g**). Data are reported as mean ± SEM. Different letters denote statistically significant differences among experimental groups (one-way ANOVA, *p* < 0.05, Dunnett’s multiple comparison test).

Histological analysis of the female ovary isolated from all experimental groups demonstrate the presence of all follicle stages, including previtellogenic- Prev-(Class I-II follicles), vitellogenic- Vit- (class III follicles), and in maturation -Mat- (class IV follicles) oocytes (Figure 2a–d). There were no significant differences in the number of Prev follicles between C and the different experimental groups. However, treatment with P in BPA+P reduced the number of BPA-induced Prev follicles to a level closer to C (Figure 2e). Similarly, treatment with BPA significantly increased the number of Vit follicles, compared to the control which was reduced following treatment with P in BPA+P (Figure 2f). Treatment with BPA did not alter the number of mature follicles compared to the control (Figure 2). The only difference observed was a significantly lower level of maturing follicles in the BPA+P compared to P treated groups (Figure 2g).

### 2.3. Real Time PCR Analisys

#### 2.3.1. Hepatic Vitellogenin (*vtg*) Transcription

In this study, we measured the hepatic mRNA levels of different *vtg* isoforms using Real-time PCR (Table 1). BPA treatment caused a decrease of *vtg1* and *vtg6* mRNA levels and an upregulation of *vtg7* form. Probiotic administration downregulated *vtg1* mRNA levels and upregulated *vtg 3*, *4*, *5*, and *7* isoforms. Surprisingly, a negative synergic action of BPA and P was observed in case of *vtg1* mRNA, which reaches lower levels in respect to those of BPA or P treatment alone, suggesting a clear antiestrogenic action. BPA and P coadministration upregulated *vtg7* transcript, but to a lower extent in respect to BPA or P group alone, although still statistically higher than in C fish. Regarding *vtg3*, the downregulation, although not statistically significant of BPA, is mitigated by P co-administration and in BPA+P group levels result similar to those measured in C fish.

Concerning *vtg4*, when co-administered, BPA antagonizes the upregulation induced by P and levels result similar to those measure in BPA and C groups. In BPA+P group *vtg5* form is significantly upregulated in respect to C and BPA fish, with levels similar to those observed in P group, suggesting an estrogenic effect of P and that this form is not BPA responsive. In BPA+P group, *vtg 6* mRNA is downregulated and levels are similar to those in BPA fish, clearly suggesting that P does not modulate this *vtg* form.

#### 2.3.2. Transcript of Genes Involved in Spermatogenesis

In this study, we measured transcript levels for a number of genes involved in the control of spermatogenesis in the zebrafish testis. Treatment with BPA significantly reduced the follicle stimulation hormone receptor, *fshr*, transcript level compared to the control. Treatment with P alone was without an effect but reversed the BPA-induced response (in BPA+P) to the control level (Table 2). Treatment with BPA significantly reduced the basal luteinizing hormone receptor, *lhcgr*, transcript level. Treatment with P alone did not change the basal *lhcgr* level and was without effect on the BPA-induced response (Table 2). Similarly, treatment with BPA significantly reduced the basal androgen receptor, *ar*, transcript level. Treatment with P was without a significant effect on the *ar* transcript level, compared to the control, but reversed the BPA-induced response to a level not different from the basal (Table 2). Treatment with BPA was without effect on the basal estrogen receptor 1, *esr1*, which was reduced following treatment with P alone and BPA+P, compared to the control (Table 2). Treatment with BPA significantly increased the basal *esr2a* transcript level, which was significantly reduced below the control and BPA level following treatments with either P alone or BPA+P (Table 2). Treatment with BPA was without effect on the basal *esr2b* transcript level, which was reduced following treatment with P alone and BPA+P, compared to the control (Table 2). While there were fluctuations, the basal membrane associated progesterone receptor 1, *pgrmc1*, transcript level was not altered significantly following treatments with BPA, P, and BPA+P (Table 2). Treatments with BPA or P alone significantly reduced the basal *pgrmc2* transcript level. Paradoxically, combined treatment with BPA and P (BPA+P) increased the *pgrmc2* transcript level to the control level (Table 2).

#### 2.3.3. Transcript of Genes Involved in Follicle Growth and Maturation

In this study, we measured transcript levels for a number of genes involved in the control of follicular growth and maturation in the isolated class III and IV follicles. Treatment with BPA alone or in combination with P, significantly increased the *fshr* transcript level compared to control in the class III follicles. Treatment with P was without effect (Table 3). Similarly, in class IV follicles, BPA-treatment significantly increased the basal *fshr* transcript level, and treatment with P was without effect on the basal or the BPA-induced response (Table 3). In class III follicles, treatment with BPA significantly increased the basal *lhcgr* transcript level, while P alone significantly reduced the basal *lhcgr* level and the BPA-induced response to the control level (Table 3). In class IV follicles, BPA treatment also significantly increased the basal *lhcgr* transcript level, but unlike class III follicles, treatment with P alone significantly increased the basal *lhcgr* transcript level. In the latter follicles, treatment with P reduced the BPA-induced response to the basal level (Table 3). In the class III follicles, treatments with either BPA or P alone significantly elevated the basal *pgrmc1* transcript level, and co-treatment with P did not alter the BPA-induced response. In the class IV follicles, treatments with either BPA or P alone did not alter the basal *pgrmc1* transcript level, but co-treatments with BPA+P significantly reduced the *pgrmc1* transcript level, compared to other groups (Table 3). In class III follicles, treatments with either BPA or P alone significantly reduced the basal *pgrmc2* transcript level well below the control. Co-treatment with both BPA and P paradoxically increased and restored the *pgrmc2* transcript level to the control level (Table 3). In class IV follicles, treatments with either BPA or P significantly reduced the basal *pgrmc2* transcript level. In the latter class of follicles, co-treatment with BPA and P resulted in a *pgrmc2* transcript level similar to P alone (Table 3).

#### 2.3.4. Multivariate Statistical Analysis

In this study, we performed unsupervised Principal Component Analysis (PCA) on three datasets obtained following the treatment of testis, class III, and class IV follicles in order to visualize the entire data set. Each point represents transcript data for each animal obtained from all treatment groups as single points corresponding to single samples and plotted into a reduced dimensional space, built with the calculated Principal Components (PCs). The position of samples into this space is given by the PC scores. The order of the PCs indicates their importance to the dataset (e.g., PC1 explains the highest amount of variation). PC loadings represent the relation between the input variables and the PCs and are used to assess how each variable contributes to a specific PC.

**Figure 3:**
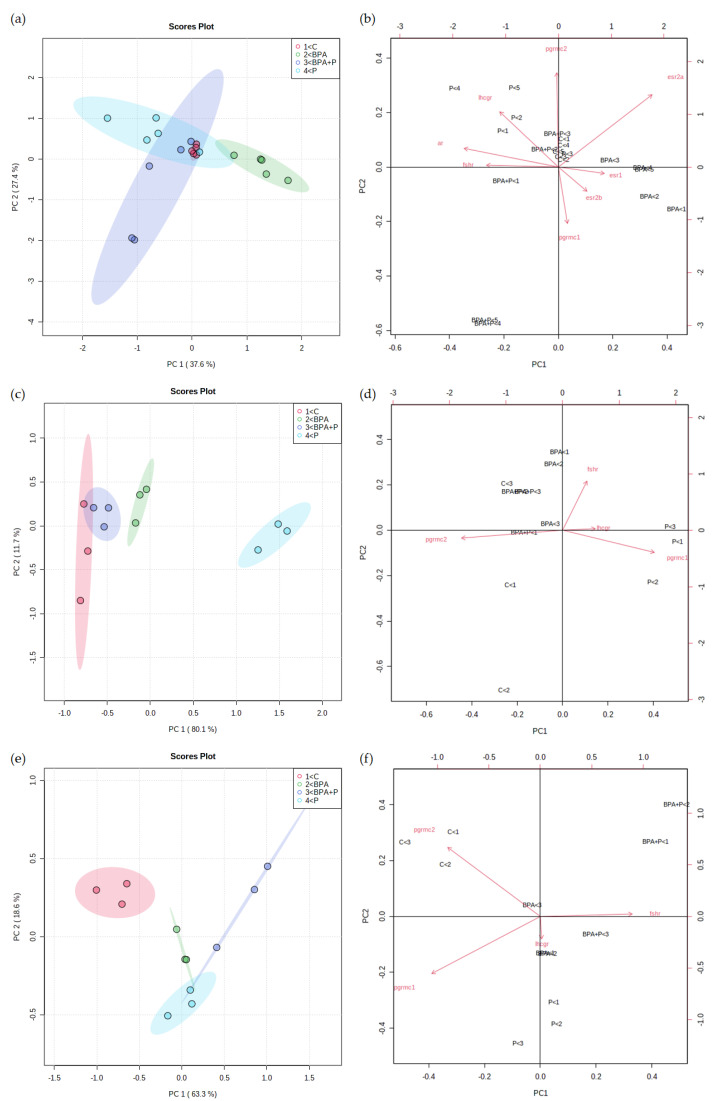
Scores plot and biplot of testis (**a,b**), class III (**c**,**d**) and class IV (**e**,**f**) follicles of mRNA levels data obtained in C (red), BPA (green), BPA+P (blue) and P (light blue) groups. (**a**,**c**,**e**) Axes show scores on PC1 and PC2; (**b**,**d**,**f**) bottom and left axes show scores on PC1 and PC2, top and right axes show loadings; red arrows show variable loadings.

PCA results on testis analysis (Figure 3a), provides satisfactory cumulative explained variance (65%) with an overlapped region between P and BPA+P, containing the C group, provided by PC1, while PC2 shows separation between BPA and all other groups. Considering class III and IV follicles analysis, Figure 3c,e show that the combination of PC1 and PC2 value provide a result in terms of cumulative explained variance for class III (91.8%) and IV (81.9%) respectively, and of group segregation. In fact, samples appeared in the plot organized in compact clusters, demonstrating the presence of no outliers. The distribution of experimental groups was consistent with the working hypothesis, considering that in class III follicles PC1 (Figure 3c), accounting for the major variance, discriminates between C and BPA and P, but not BPA+P, while PC2 did not show a separation among groups. In class IV oocytes, PC1 discriminates between C and all treatments, while PC2 does not show separation between C and BPA+P (Figure 3e).

In general terms, data demonstrate the ability of P, when co-administered with BPA, to bring the BPA+P group closer to P, as observed in males or in class IV follicles or to C as seen in class III ones. These data suggest the ability of P to counteract BPA effect, showing the mitigating role of this probiotic formulation at a gonadal level.

The biplot shows the analysis of loadings together with the PCA, making it possible to understand the variable’s contribution to model building. Red arrows display the loading of each variable. Male PCA present *fshr*, *ar*, and *esr1*, contributing to PC1, *pgrmc 1* and *pgrmc2* have an effect on PC2, while *lhcgr*, *esr2a*, and *esr2b* have an impact on both PCs (Figure 3b).

PC1 big variance is explained by the fact that most of the variable presents the large effect of this component: *lhcgr*, *pgrmc1*, and *pgrmc2* in class III oocytes (Figure 3d), and *fshr* and *pgrmc1* in class IV oocytes (Figure 3f), while in this class *pgrmc2* accounts for separation in both PC1 and PC2. Only *fshr* in class III follicles, and *lhcgr* in class IV follicles contribute to PC2, with *lhcgr* showing little impact.

## 3. Discussion

The beneficial role of SLAb51 administration was previously demonstrated in different animal models: and enhancement of specific immune functions associated to changes of intestinal microbiota was observed in healthy dogs [24], while in mice models affected by Alzheimer’s disease, a reduction of brain oxidative damages [25] was described.

This study presents the first results related to the role of SLAb51 in female and male zebrafish reproduction and evidenced its ability to mitigate BPA reproductive toxicity [9,26], in some cases antagonizing its well-known disruptive actions. Reproduction, indeed, is controlled by a delicate balance that is established between endocrine and paracrine factors [27,28] and a set of genes are involved in the intricate processes leading to the production of gonadal steroids that facilitate the production of viable gametes. Consistent with previous observations in rats [29,30], the results herein obtained in fish exposed to BPA clearly show an alteration of spermatogenesis and confirm a previous study in zebrafish [26]. These last authors demonstrated that the adverse actions of BPA are in part mediated by disrupting the endocannabinoid system (ECS) functioning by competing with its endogenous ligands. The correct ECS functioning is, in fact, essential for the normal progression of the male and female reproductive processes and the loss of anandamide binding is responsible for the observed decrease in spermatogonia numbers. Aside from interacting with the ECS, BPA can also decrease GnRH levels [31], in turn, disrupt the production of gonadotropins and testosterone, leading to a reduction of spermatogenesis [32]. In this study, we observed a BPA-mediated reduction of both gonadotropin receptor mRNA levels, which could potentially affect the FSH activity needed for conversion of spermatogonia A into B, and in case of LH, affect the final spermiogenic phase [33]. Regarding this last aspect, since the whole spermatogenesis in zebrafish lasts 28 days, we cannot exclude that longer exposure to BPA may also affect spermatozoa numbers. In addition, BPA could also exert antiandrogenic effects as indicated by the observed decrease in *ar* mRNA, with the possible consequence of reducing the activity of androgens such as T and 11-ketosterone [34,35]. In this context, the production of androgens may also be caused by the decrease of FSH [36]. Moving to SLAb51, the present results demonstrate its potential beneficial actions on spermatogenesis, either when administered alone or with BPA. In BPA+P group, P counteracts BPA-induced *fshr* mRNA downregulation, and this could be responsible for the observed increase in the number of spermatogonia. The increase of *fshr* transcript can be associated to an increase of FSH, as previously demonstrated in zebrafish [33] or in eels fed *Lactobacillus rhamnosus* [37].

In females, the number of vitellogenic oocytes observed following BPA exposure confirms previous data [26,38] indicating the estrogen-like activity of this contaminant thus upregulating vitellogenin transcript levels [39,40]. Similarly, as previously observed in fish treated with *L. rhanmosus* [14,16], also SLAb51 administration stimulated certain forms of *vtg*. Focusing on vitellogenesis, in oviparous vertebrates this process starts in the liver and is triggered by gonadal estrogens [41,42]. Zebrafish contain 8 different forms of vitellogenin genes [43] encoding for 3 proteins including type I (*vtg1*, *vtg4*, *vtg5*, *vtg6*, and *vtg7*), type II (*vtg2* and *vtg8*) and type III (*vtg3*) [44], which differently contribute to the embryonic morphogenesis, hatching, larval kinetics and survival (*vtg1*, *3*, *4*, and *5*) or provide homeostatic regulation of total *vtg* levels (*vtg7*) [45,46]. In the present study, the observed increase in *vtg7* mRNA levels in all groups suggests the activation of a compensatory mechanism due to the alterations of the other gene forms. An increase of *vtg7*, in fact, has been observed both at the mRNA and protein levels in knock-out zebrafish phenotypes [46]. The role of *vtg* subtypes may be different among fish species. Differently from what is observed in this study, in seabream fed with BPA-contaminated feed, an increase of *vtga* and *b,* corresponding to zebrafish type 1 and 2 forms was induced. The observed increase in transcription of *vtg* forms following treatment with SLAb51 is consistent with the results obtained in fish receiving *L. rhamnosus*, possibly involving changes in neuropeptides and metabolic signals, thus suggesting its positive effect on reproduction as a food additive [47]. The present results also demonstrate the ability of SLAb51 to mitigate BPA effects on *vtg 3* and *5* forms, which are crucial for fertility and embryo development. As previously described in catfish [48] or in zebrafish larvae [49] exposed to similar BPA concentrations, also in the present study, BPA exposure determined an increase of gonadotrophin receptor mRNA in both follicle classes, while data in the whole ovary reported that the same dose did not affect *fshr* levels, and significantly inhibits the transcription of *lhcgr* [8]. The present results demonstrate that SLAb51 can differently modulate *fshr* and *lhcgr* mRNA, and counteract BPA effects.

P differently modulates *fshr* and *lhcgr* mRNA, and in class III and IV follicles, its ability to contrast BPA effect was evidenced: mRNA levels were closer to those of C fish. However, thr *fshr* transcript trend does not reflect the number of vitellogenic oocytes, suggesting that BPA and P most likely may affect not only gene transcription but also protein synthesis.

In addition, BPA and SLAb51 do not affect maturation process which is featured by the localization of progesterone receptors on class III oocyte membrane, which are therefore defined as maturationally competent [50]. Previous studies have shown that *pgrmc1* and *2* mRNA levels vary during follicle development, increasing in later stages. Zebrafish pgrmc1 ^−/−^ show a reduction of fecundity and fertility [51] and similarly, in female mice, conditional ablation of *pgrmc1* results in reduced fertility, while elimination of *pgrmc2* causes premature reproductive senescence [52]. In class III oocytes isolated from fish exposed to BPA or P, an increase in the expression of form 1 and a down-regulation of form 2 was observed and therefore since they are both involved in the maturation process [53], this could result in the lack of increase of maturing oocytes, despite the higher number of vitellogenic ones. In BPA+P group, mRNA transcripts suggest the ability of P to antagonize BPA as clearly demonstrated by levels similar to C ones. The scenario herein described is very similar to that observed in a study on zebrafish co-exposed to *L. rhamnosus* and perfluorobutanesulfonate (PFBS) [23], where co-exposure almost ceased the fecundity, which was accompanied by disturbances in sex hormones and oocyte maturation in females [54], in contrast, in males, probiotic additive efficiently antagonized the estrogenic activity of PFBS. Nevertheless, these authors demonstrated the antagonistic interaction between PFBS and *L. rhamnosus* regarding the metabolic activities along the microbe, gut and liver axis [55] with an efficient mitigation of lipid [56] and glucose [57] metabolic disorders associated with PFBS exposure, highlighting the potential values of probiotic bacteria used to protect the aquatic ecosystem. Different bacteria strains have been proven so far to promote the degradation of specific environmental pollutants that result in being harmful to organisms while increasing host resistance and resilience [58,59] This was clearly demonstrated in a trial where the toxicity of triclosan (TCS) was alleviated by feeding zebrafish *Lactobacillus plantarum ST-III*; dietary probiotic administration alleviated the intestinal metabolic syndromes and neurodegenerative diseases resulting from exposure to TCS, through modifying the gut flora [60].

In conclusion, these results present the first preliminary data supporting the hypothesis that SLAb51 can antagonize/mitigate some BPA toxic action in zebrafish and likely in other vertebrate species. The findings also suggest the ability of SLAb51 to positively interact with spermatogenesis, while regarding oocyte growth and maturation further investigation are needed. Changes of probiotic concentrations or trial duration could be useful to clarify the SLAb51 reproductive effects also in female zebrafish. This would help in building up a comprehensive scenario regarding Slab51 effects in zebrafish often used as aquatic species model, in the light of supporting aquaculture practices or within a bioremediation contest. In the last years, interest on this last aspect is in fact increasing and the use of bacteria is considered a good strategy to guarantee sustainability, as it has a relatively low cost and can be applied in different ecosystems, causing minimal impact to the environment.

On this regard, the results obtained in this study, together with those obtained by other authors showing the possible role of probiotics in counteracting the toxic effect of different EDCs on several physiological process should encourage further research in order to optimize the use of probiotics to mitigate the effect of the many toxicants ubiquitously present worldwide.

## 4. Materials and Methods

### 4.1. SLAb51^®^ (SivoMixx^®^)

SLAb51^®^ (SivoMixx^®^, Ormendes SA, Jouxtens-Mézery, CH, Switzerland) is a commercial multi-strain probiotic containing 200 billion lactic acid bacteria per 1.5 grams of product, comprised of the following strains: *Streptococcus thermophilus DSM 32245*, *Bifidobacterium lactis DSM 32246*, *Bifidobacterium lactis DSM 32247*, *Lactobacillus acidophilus DSM 32241*, *Lactobacillus helveticus DSM 32242*, *Lactobacillus paracasei DSM 32243*, *Lactobacillus plantarum DSM 32244*, and *Lactobacillus brevis DSM 27961*.

### 4.2. Animal Treatment

A total of 80 adult zebrafish (40 male and 40 female) (*D. rerio*, AB wild-type strain) were divided into 8 10-L aquaria (10 fish/tank) with oxygenated water under controlled conditions (28.0 ± 0.5 °C) and maintained on a 14/10 h light/dark cycle. They were fed twice a day commercial food (Vipagran; Sera, Loessnitz, Germany). The experiment was set up in duplicate as follows:

C: control fish fed twice a day with commercial food (Vipagran; Sera, Loessnitz, Germany)

BPA: fish were fed commercial food and were exposed to 10 μg/L BPA (98% analytical purity, Sigma-Aldrich, Milano, Italy)

BPA+P: fish were exposed to 10 μg/L BPA and fed commercial food supplemented with SLAb51 at a final concentration of 10^9^ CFU/g

P: fish were fed commercial food fish and received a dietary supplementation of SLAb51 at a final concentration of 10^9^ CFU/g

All groups were sampled after 4 weeks of treatment.

At the end of the trial, fish were lethally anesthetized with 500 mg/L MS-222 (3-aminobenzoic acid ethyl ester, Sigma Aldrich) buffered to pH 7.4. Livers were stored at −80 °C for molecular analysis. Testis and ovaries were dissected out and divided as follows: 5 samples were fixed in Bouin’s fixative for histology. The remaining 5 testis were stored at −80 °C, while ovaries were teased into separate follicles using transfer pipettes (Semco Scientific Corp., San Diego, CA, USA) without trypsinization; thereafter, follicles were separated into different maturation stages according to their diameters, as previously described [61,62] and class III and IV follicles were collected and stored at −80 °C for molecular analysis.

### 4.3. Fish Fertility

Reproductive performances of each experimental group were assayed in spawning tanks under standard conditions as previously described [8]. Starting on the 8th day of treatment, male and female zebrafish from the 4 groups were crossed and fertility was determined during the following 28 days. Fertilized eggs were counted, and the fertility rate was calculated as the mean ± standard deviation (SD) of fertilized egg number/female/day from the 8th to the 21st day of treatment.

### 4.4. Gonad Histology and Image Analysis

Hystological analyses were performed on 5 ovaries and testis for each experimental group according to Forner-Piquer et al., 2017 [63].

### 4.5. RNA Extraction and cDNA Synthesis

For each experimental group, total RNA was extracted from 5 female livers and 5 testis, and from 3, classes III and IV pools, containing 50 follicles each, using RNAeasy^®^ Minikit (Qiagen, Milano, Italy). RNA quality assessment and cDNA synthesis were performed as previously described [61].

### 4.6. Real-Time PCR

qRT-PCRs were performed with SYBR green in an CFX thermal cycler, as previously described [26]. Ribosomal protein 13 (*rpl13*) and ribosomal protein 0 (*rplp0*) mRNAs were used as internal standards in each sample in order to standardize the results by eliminating variation in mRNA and cDNA quantity and quality. Primer sequences, GenBank accession numbers and primer efficiency of the examined genes are reported in Table 4.

mRNA levels of each target gene analyzed were calculated using the Pfaffl method [64], relative to the geometric mean of the two reference genes once demonstrated they were stably expressed by the geNorm algorithm, both implemented in the Bio-Rad CFX Manager 3.1. software. Modification of gene expression among the experimental groups is reported as relative mRNA abundance (arbitrary units). Primers were used at a final concentration of 10 pmol/mL

### 4.7. Statistical Analysis

All the data were analyzed by One-Way ANOVA followed by Dunnett’s multiple comparison test. When the collected data was expressed in percentage, arcsin transformation was conducted before ANOVA. All statistical analyses were performed using the statistical soft- ware package Prism5 (GraphPad Software, Inc., San Diego, CA, USA) with significance accepted at *p* < 0.05. Unsupervised Principal Component Analysis (PCA) were performed using Metaboanalyst 5.0 online platform (University of Aberta, Edmonton, AB, Canada). Data sets were created using genes with *p*-value < 0.05, and underwent normalization, using normalization by median, transformation, with log transformation, and scaling, using pareto scaling. A 2D score plot was generated, plotting the first two principal component, to show separation among groups based on gene expression profile.

## Figures and Tables

**Table 1:** Hepatic *vtg* mRNA expression values in the different experimental groups. Data are reported as means ± SD. Different letters indicate statistically significant variations among groups. (one-way ANOVA followed by Dunnett’s multiple comparison test *p* < 0.05).

Female Liver	C	BPA	BPA+P	P
*vtg1*	7.89 ± 1.05 ^(a)^	3.25 ± 1.20 ^(b)^	1.82 ± 0.73 ^(c)^	5.36 ± 0.87 ^(d)^
*vtg2*	1.69 ± 0.20 ^(a)^	2.00 ± 0.82 ^(a)^	1.51 ± 0.49 ^(a)^	2.52 ± 0.35 ^(a)^
*vtg3*	4.52 ± 0.37 ^(a,b)^	3.69 ± 0.91 ^(a)^	5.03 ± 0.59 ^(b)^	6.49± 0.49 ^(c)^
*vtg4*	3.51 ± 1.20 ^(a)^	3.26 ± 0.52 ^(a)^	2.78 ± 0.87 ^(a)^	8.66 ± 0.50 ^(b)^
*vtg5*	2.10 ± 0.63 ^(a)^	2.37 ± 0.35 ^(a)^	4.41 ± 0.65 ^(b)^	5.08 ± 0.59 ^(b)^
*vtg6*	8.39 ± 0.32 ^(a)^	3.29 ± 0.96 ^(b)^	3.46 ± 0.68 ^(b)^	9.39 ± 0.89 ^(a)^
*vtg7*	2.59 ± 0.44 ^(a)^	11.42 ± 0.84 ^(b)^	6.08 ± 0.60 ^(c)^	23.02 ± 0.79 ^(d)^

**Table 2:** mRNA expression values of genes regulating spermatogenesis in the testis of the different experimental groups. Data are reported as means ± SD. Different letters indicate statistically significant variations among the groups (one-way ANOVA followed by Dunnett’s multiple comparison test, *p* < 0.05).

Testis	C	BPA	BPA+P	P
*fshr*	10.77 ± 2.22 ^(a)^	2.82 ± 0.27 ^(b)^	7.3 ± 1.82 ^(a)^	9.72 ± 4.67 ^(a)^
*lhcgr*	6.9 ± 0.69 ^(a)^	3.92 ± 0.94 ^(b)^	3.45 ± 1.45 ^(b)^	5.6 ± 2.14 ^(a,b)^
*ar*	5.15 ± 0.76 ^(a)^	1.88 ± 0.69 ^(b)^	3.57 ± 2.10 ^(a,b)^	2.81 ± 1.64 ^(a,b)^
*esr1*	8.41 ± 0.61 ^(a)^	8.57 ± 1.20 ^(a)^	3.4 ± 1.12 ^(b)^	3.03 ± 1.10 ^(b)^
*esr2a*	5.31 ± 0.84 ^(a)^	8.11 ± 1.52 ^(b)^	2.05 ± 0.70 ^(c)^	2.12 ± 1.41 ^(c)^
*esr2b*	6.17± 1.23 ^(a)^	4.03 ± 1.2 ^(a,b)^	2.05 ± 0.41 ^(b,c)^	1.1 ± 0.32 ^(c)^
*pgrmc1*	5.60 ± 1.37 ^(a,b)^	7.96 ± 2.06 ^(a)^	5.52 ± 1.89 ^(a,b)^	2.63 ± 0.93 ^(b)^
*pgrmc2*	7.34 ± 2.06 ^(a)^	3.99 ± 1.50 ^(b)^	5.64 ± 1.14 ^(a,b)^	3.46 ± 1.17 ^(b)^

**Table 3:** mRNA expression values of genes regulating follicle growth and maturation in class III (a) and IV (b) follicles. Data are reported as means ± SD. Different letters indicate statistically significant variations among groups (one-way ANOVA followed by Dunnett’s multiple comparison test, *p* < 0.05).

**(a)**				
**Class III Follicles**	**C**	**BPA**	**BPA+P**	**P**
*fshr*	1.85 ± 0.80 ^(a)^	5.15 ± 0.85 ^(b)^	4.03 ± 0.12 ^(b)^	1.18 ± 0.25 ^(a)^
*lhcgr*	3.39 ± 0.82 ^(a)^	5.63 ± 0.19 ^(b)^	3.32 ± 0.4 ^(a)^	1.41 ± 0.57 ^(c)^
*pgrmc1*	4.95 ± 0.07 ^(a)^	6.91 ± 0.20 ^(b)^	6.33 ± 1.19 ^(a,b)^	11.70 ± 0.02 ^(c)^
*pgrmc2*	40.04 ± 5.60 ^(a)^	19.85 ± 1.54 ^(b)^	38.75 ± 1.90 ^(a)^	1.25 ± 0.36 ^(c)^
**(b)**				
**Class IV Follicles**	**C**	**BPA**	**BPA+P**	**P**
*fshr*	1.72 ± 0.68 ^(a)^	4.43 ± 0.77 ^(b)^	4.05 ± 0.24 ^(b,c)^	3.02 ± 0.49 ^(a,c)^
*lhcgr*	4.30 ± 0.27 ^(a)^	6.65 ± 0.36 ^(b)^	4.44 ± 0.29 ^(a)^	6.01 ± 0.47 ^(b)^
*pgrmc1*	3.41 ± 0.77 ^(a)^	4.22 ± 0.69 ^(a)^	1.65 ± 0.92 ^(b)^	4.11 ± 0.84 ^(a)^
*pgrmc2*	33.21 ± 1.82 ^(a)^	24.79 ± 4.68 ^(b)^	15.37 ± 0.66 ^(c)^	16.45 ± 0.35 ^(c)^

**Table 4:** Primer list.

Gene Name	Symbol	Forward	Reverse	Source
Vitellogenin 1	*vtg 1*	GATTAAGCGTACACTGAGACCA	AGCCACTTCTTGTCCAAATACT	[61]
Vitellogenin 2	*vtg 2*	TGCCGCATGAAACTTGAATCT	GTTCTTACTGGTGCACAGCC	[61]
Vitellogenin 3	*vtg 3*	GGGAAAGGATTCAAGATGTTCAGA	ATTTGCTGATTTCAACTGGGAGAC	[61]
Vitellogenin 4	*vtg 4*	TCCAGACGGTACTTTCACCA	CTGACAGTTCTGCATCAACACA	[61]
Vitellogenin 5	*vtg 5*	ATTGCCAAGAAAGAGCCCAA	TTCAGCCTCAAACAGCACAA	[61]
Vitellogenin 6	*vtg 6*	TTTGGTGTGAGAACTGGAGG	CCAGTTTGTGAGTGCTTTCAG	[61]
Vitellogenin 7	*vtg 7*	TTGGTGTGAGAACTGGAGGA	TTGCAAGTGCCTTCAGTGTA	[61]
Luteinizing hormone receptor	*lhcgr*	GGCGAAGGCTAGATGGCACAT	TCGCAATCTGGTTCATCAATA	[8]
Follicle stimulating hormone	*fshr*	GGATTCTTCACCGTCTTCTCC	TGTAGCTGCTCAACTCAAACA	[8]
Estrogen recptor 1	*esr1*	GGTCCAGTGTGGTGTCCTCT	AGAAAGCTTTGCATCCCTCA	[8]
Estrogen receptor 2a	*esr2a*	TTGTGTTCTCCAGCATGAGC	CCACATATGGGGAAGGAATG	[8]
Estrogen receptor 2b	*esr2b*	TAGTGGGACTTGGACCGAAC	TTCACACGACCACACTCCAT	[8]
Membrane-associated progesterone receptor component 1	*pgrmc1*	CGGTTGTGATGGAGCAGATT	AGTAGCGCCAGTTCTGGTCA	[8]
Membrane-associated progesterone receptor component 2	*pgrmc2*	ACAACGAGCTGCTGAATGTG	ATGGGCCAGTTCAGAGTGAG	[8]
Androgen receptor	*ar*	ACTGGGACCGAATAAAGCCC	ATGTAATCGCAGCCGGAGAC	[8]
Ribosomal protein 13	*rpl13*	TCTGGAGACTGTAAGAGGTATGC	AGACGCACAATCTTGAGAGCAG	[8]
Ribosomal protein large, P0	*rplp0*	CTGAACATCTCGCCCTTCTC	TAGCCGATCTGCAGACACAC	[8]
